# Effects of cocaine and levamisole (as adulterant) on the isolated perfused Langendorff heart

**DOI:** 10.1007/s00414-020-02300-5

**Published:** 2020-05-06

**Authors:** A. Gartz, E. Pawlik, J. Eckhardt, St. Ritz-Timme, R. Huhn, F. Mayer

**Affiliations:** 1 Institute for Legal Medicine, University Hospital Düsseldorf, Moorenstr. 5, 40225 Düsseldorf, Germany; 2 Departement of Anaesthesiology, University Hospital Düsseldorf, Moorenstr. 5, 40225 Düsseldorf, Germany

**Keywords:** Cocaine, Levamisole, Langendorff-heart, Cardiac function, Heart rate, Cardiac flow, Left ventricular pressure

## Abstract

Cocaine-related deaths occur regularly in forensic routine work. In cases in which the detected concentration of cocaine is rather low and other causes of death apart from intoxication can be ruled out, the question arises if adulterants of cocaine might have played a crucial role. In the present study, cardiac effects of cocaine, of the adulterant levamisole and of mixtures of both were evaluated using the isolated perfused Langendorff heart. While exposed to the substances, functional parameters heart rate, left ventricular pressure and coronary flow were documented. Relevant alterations of these parameters were found for cocaine as well as for levamisole. Exposing the hearts to a mixture of both resulted in a combination of these effects; the emergence of new alterations or an obvious aggravation were not detected. Nevertheless, the results imply that the consumption of cocaine adulterated with levamisole bares an increased risk for cardiac complications, especially in the presence of preexisting cardiac pathologies.

## Introduction

Cocaine is one of the most frequently consumed drugs, and the number of consumers is still rising. Columbia is the largest producer of coca worldwide. In 2016, the UNODC, the United Nation Office on Drugs and Crime, described an increase of the coca cultivation area of 52% to 146,000 ha with an estimated increase of cocaine production of 34% to 866 t per year [1]. In 2016, Germany faced more than 13.000 crimes related to cocaine which means a plus of 20.7% [2]. In the same year, approximately 1300 cases of death in Germany were related to the consumption of drugs. This number has risen for the fourth time in succession [3]. Five percent of drug fatalities were caused by cocaine or a combination of cocaine and other drugs [4].

A lot of studies that investigated the physiologic effects of cocaine as well as reviews of the respective research findings, e.g. Karch’s Pathology of Drug Abuse [5], have been published. Cocaine acts as a stimulant, making the consumer feel powerful, awake, cheerful and more productive. It is also known to influence cardiovascular function parameters such as heart rate and blood pressure and to cause constrictions of coronary vessels, even coronary artery spasms. Chronic consumption also causes changes in structure and function of heart and blood vessels like arterial stiffness and increase of left ventricular mass [6]. Cardiovascular complications like cardiac hypertrophy, obstructive small vessel disease and premature coronary sclerosis are the main reasons for cocaine-related sudden deaths [7].

Though street cocaine purity has increased over the last 10 years, the share of cocaine has increased from 40 to 70% [8], the use of adulterants is still common practice. Frequently used adulterants are levamisole (anthelmintic), diltiazem (calcium channel blocker), local anaesthetics and hydroxyzine (antihistaminic) [9]. These adulterants are supposed to imitate the effect of cocaine and to prolong and intensify the high, but their main purpose is to increase the profit of drug producers and dealers. In 2003, cocaine adulterated with levamisole was discovered in the USA for the first time by the DEA (Drug Enforcement Administration) [10] and since 2005 there were multiple reports on the presence of levamisole in confiscated cocaine [11]. In 2014, a study revealed that 70% of street cocaine was adulterated with levamisole [12]. It has been suggested that levamisole might act synergistically with cocaine in producing a longer and intensified ‘high’ [13]. With a view to cardiac function, levamisole can cause right heart failure [14]. Furthermore, effects on heart rate have been observed including severe bradycardia, tachycardia and ventricular fibrillation, as well as cardiac dysrhythmias [15]. In veterinary medicine, levamisole is used as an anthelmintic for artiodactyls. It is metabolized to aminorex which has amphetamine-like effects [16]. In the 1970s, levamisole was forbidden in human medicine because of increasing numbers of pulmonary hypertension that have been attributed to a levamisole-containing appetite suppressor [17]. Also, cases of pulmonary vasculitis have been described after consumption of levamisole adulterated cocaine [18]. It is presumed that levamisole is absorbed in the gastrointestinal tract and metabolized to aminorex in the liver; however, this is not completely clear [19, 20]. When establishing a method to detect levamisole and its metabolites in human plasma, Hess et al. found rather low concentrations of aminorex after ingestion of levamisole [21]. It is also unclear to which extend each levamisole and aminorex contribute to the described effects on the cardiovascular system.

In forensic routine work, cases of death related to the consumption of cocaine occur regularly. However, in some of these cases, the measured cocaine concentrations are quite low and cannot explain death without further ado. If no other plausible cause of death can be found, the question arises, if influences of one or more adulterants, especially effects on heart function, might have played a crucial role and contributed to cardiac failure.

With the present study, we wanted to address this question. We worked with an animal model, the isolated perfused Langendorff heart, in order to find out if levamisole might cause cardiac adverse effects that may play a role in sudden death after the consumption of street cocaine. The Langendorff system offers the possibility to observe heart function under the influence of one or more substances. Changes of functional parameters like intra-ventricular pressure, heart rate and coronary flow can be monitored as well as changes of the electrocardiogram. Post mortem harvested hearts of rats were exposed to cocaine and levamisole as pure substances, as well as to defined mixtures of both. With a view to the above mentioned scenario, we worked with rather low amounts of cocaine and levamisole respectively.

The study aimed on answering the following questions:

- Which effects do cocaine and levamisole as single substances have on the function of the isolated perfused Langendorff heart?
- Is there an alteration, maybe even a potentiation of the observed effects when the hearts are exposed to a mixture of both substances?

## Material and methods

All experiments were performed in accordance with the German legislation on protection of animals and the Guide for the Care and Use of Laboratory Animals published by the US National Institutes of Health (NIH Publication No. 85-23, revised 1996). The protocol for the Langendorff system was approved by the local Animal Ethics Committee (project no. O 27/11).

### The isolated Langendorff heart

The preparation of the rats’ hearts was to a great extend performed as described before [22]. White male Wistar rats aged 2 to 3 months and weighing 250 to 400 g were used for this study. The rats were anaesthetised by intraperitoneal injection of 29 ng pentobarbital and 1000 IE heparin. Absence of reflexes was tested to verify deep sedation. Rats were decapitated and the hearts were excised immediately and mounted on the Langendorff system. The hearts were perfused at constant pressure (around 80 mmHg) with a modified Krebs-Hensleit buffer solution. Oxygenation was realized with a carbogen mixture consisting of 95% O_2_ and 5% CO_2_. All hearts underwent a stabilization period of 20 min. Left ventricular pressure (LVP) was measured with a balloon in the left ventricle. Electric activity (electrocardiogram - ECG) and heart rate were measured by placing one electrode at the apex, one electrode left to the left coronary artery (LCR) near the left atrial appendage and one electrode at the aorta. Coronary flow (CF) was measured by collecting and weighing the buffer solution that passed through the heart in 1 min.

The modified Krebs-Henseleit buffer solution was prepared as follows:

- 118 mM sodium chloride (VWR chemicals prolabo)
- 4.7 mM potassium chloride (Fluka)
- 1.2 mM magnesium sulphate hepta-hydrate (Sigma-Aldrich)
- 1.2 mM potassium dihydrogen phosphate (Merck)
- 25 mM sodium hydrogen carbonate (Roth)
- 0.5 mM Ethylenediaminetetraacetic acid (Roth)
- 11 mM d-glucose (VWR life science and Roth)
- 1 mM l-lactic acid sodium salt (Serva)
- 2.25 mM calcium chloride (Merck).

The data were digitalized using an analogue to digital converter (PowerLab/8SP, ADInstruments Pty Ltd., Castle hill, Australia) at a sampling rate of 500 Hz, and they were recorded continuously on a personal computer using Chart for Windows v5.0 (ADInstruments).

After the experiments all hearts were fixed in formalin, tissue sections were prepared and stained with hemtaoxylin and eosin (H&E).

### Study group

The study group contained 42 hearts in total. All substances (cocaine, levamisole, mixture of cocaine and levamisole) were administered by a perfusor with a rate of 60 mL/h over 5 minutes (“exposure-period”). During the application of the substances, LVP and heart rate were measured every minute, CF in the first and in the fifth minute. After the application of the substances, we aimed on monitoring the hearts for another 2 h and capture all parameters after 1, 5, 10, 20, 30, 60, 90, and 120 min (“recovery-period”). However, due to a sharp decline of their function some hearts had to be removed earlier.

Stock solutions of each cocaine and levamisole in acetonitrile (ACN) with concentrations of 100 μg/mL and 1 mg/mL were prepared. Cocaine was dissolved in acetonitrile to prevent its spontaneous hydrolysis to benzoylecgonine (BZE). Different quantities of these stock solutions were added to 5 mL of Krebs-Hensleit buffer in a 50-mL perfusor syringe in order to expose the study hearts to different amounts of the substances.

Multiple measurements were performed especially for cocaine, the numbers of which are shown in brackets. Mixture ratios were chosen according to analytical results of confiscated cocaine.

Twenty-three hearts were exposed to cocaine in the following quantities: 0.5 μg (2), 0.7 μg (2), 0.9 μg (2), 1 μg (2), 1.5 μg (3), 2 μg (2), 2.5 μg (2), 3 μg (2), 3.5 μg (3), 5 μg (1) and 50 μg (2).

Ten hearts were exposed to levamisole in quantities of 0.3 μg (1), 0.4 μg (1), 0.5 μg (2), 0.7 μg (1), 0.9 μg (2), 1 μg (1), 1.5 μg (1) and 5 μg (1).

Defined mixtures of cocaine and levamisole were prepared and added to ACN in a concentration of 100 μg/mL. Different quantities of this stock solution were then added to 5 mL of Krebs-Hensleit buffer in a 50-mL perfusor syringe so that the hearts in the Langendorff system were exposed to either 0.7 μg or 2 μg cocaine and the respective amount of levamisole deriving from ratios of 60:40, 70:30, 80:20, 90:10 (cocaine: levamisole). One heart was exposed to 5 μg of cocaine in a mixture with levamisole at the ratio of 80:20.

### Control group

The control group comprised 4 hearts. One heart was mounted on the Langendorff-system and exposed to five millilitres of buffer solution over 5 min by a perfusor with a rate of 60 mL/h in addition to the basic amount of buffer solution needed for the experiment. Three hearts were exposed to 0.1 mL of ACN administered by a perfusor with a rate of 60 mL/h over 5 min in 5 ml of buffer.

LVP, heart rate and CF were captured after 1, 5, 10, 20, 30, 60, 90 and 120 min. One heart treated with an additional amount of ACN had to be removed after 90 min due to a rapid decline of its function.

## Results

For some amounts of levamisole and especially cocaine multiple measurements were performed (see above). Figures show mean values of the results for each cocaine/levamisole quantity for a clearer depiction, since obvious deviations of the measured values were found only in single cases which are described separately. Figures are presented as stacked line charts in order to make them clearer. This has to be kept in mind when comparing the graphs with the values presented in the text.

### Heart rate

**Fig. 1:**
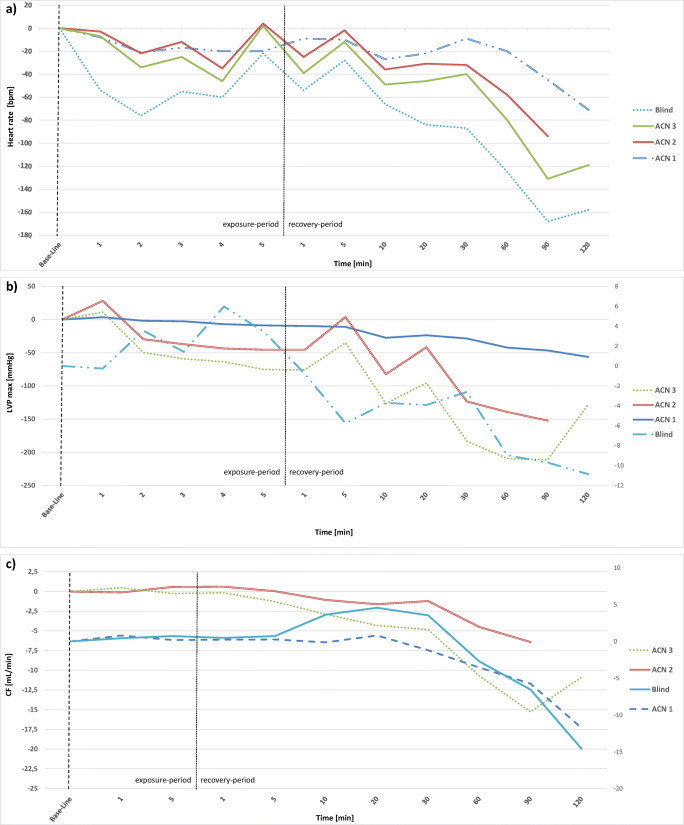
Results of control hearts, presented as stacked line charts: **a** heart rate, **b** cardiac flow, **c** left ventricular pressure. Hearts were either exposed to an additional amount of buffer solution (Blind) or acetonitrile (ACN). Value “0” is equivalent to the individual base line of each heart after the stabilization period. The first 5 min represent the exposure period, the following minutes 1 to 120 represent the recovery period

**Fig. 2:**
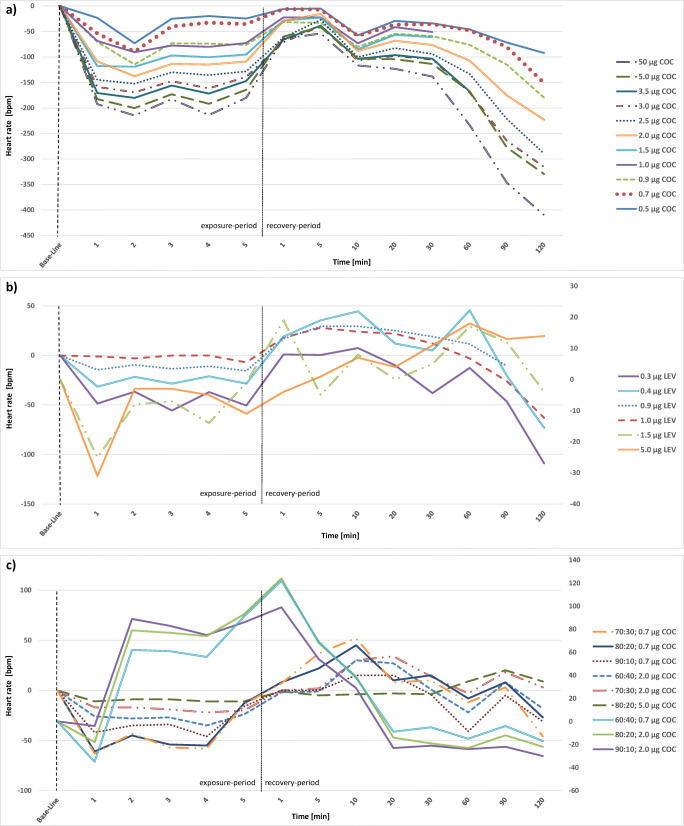
Alterations of heart rate in study hearts, presented as stacked line charts: **a** cocaine hearts (COC), **b** levamisole hearts (LEV), **c** mixture hearts cocaine: levamisole with either 0.7 μg or 2.0 μg cocaine (COC). Value “0” is equivalent to the individual base line of each heart after the stabilization period. The first 5 min represent the exposure period, the following minutes 1 to 120 represent the recovery period. Mean values of several individual measurements are shown for a clearer depiction

Hearts in the Langendorff system showed varying heart rates after the stabilization period between 250 and 450 bpm. Changes of heart rate during the exposure to the evaluated substances and the following recovery-period are expressed as the deviation from these individual “base lines” and are presented in Figs. 1 and 2.

The heart rate of control hearts (Fig. 1a) showed a decrease of 21 to 47 bpm during the exposure to buffer or ACN and restabilized until minute 5 around the individual baseline value. During the recovery period, the heart rate slightly decreased by 39 to 71 bpm until the end of the experiment.

During the exposure to cocaine (Fig. 2a) study hearts mainly presented a deceleration of the heart rate by 6 to 63 bpm followed by a stabilization around the baseline values. Merely for the hearts exposed to 1 μg cocaine an acceleration of the heart rate by 24 bpm during the exposure-period could be observed. The results for the ongoing measurement are slightly inhomogeneous but overall, a further deceleration of the heart rate by of 13 to 88 bpm was observed.

While exposed to levamisole (Fig. 2b) most hearts presented a deceleration of the heart rate by 7 to 27 bpm. Single hearts showed only little changes (1 μg levamisole) or an acceleration of the heart rate by 10 bpm (1.5 μg levamisole). In the recovery period, the observed alterations were very inhomogeneous. Accelerations as well as decelerations were observed at different points of time, a uniform tendency could not be determined.

Administration of a cocaine and levamisole mixture (Fig. 2c) caused a deceleration of heart rate by 3 to 27 bpm. Afterwards the heart rate accelerated by 4 to 25 bpm before once again showing a decline by 9 to 41 bpm at the end of the recovery period. Only the heart exposed to a mixture at a ratio of 90:10 (cocaine:levamisole) with 2 μg cocaine presented an acceleration of the heart rate during the exposure-period by 99 bpm, followed by a sharp decline by 122 bpm in the recovery period until the 20th minute.

### LVP_max_

**Fig. 3:**
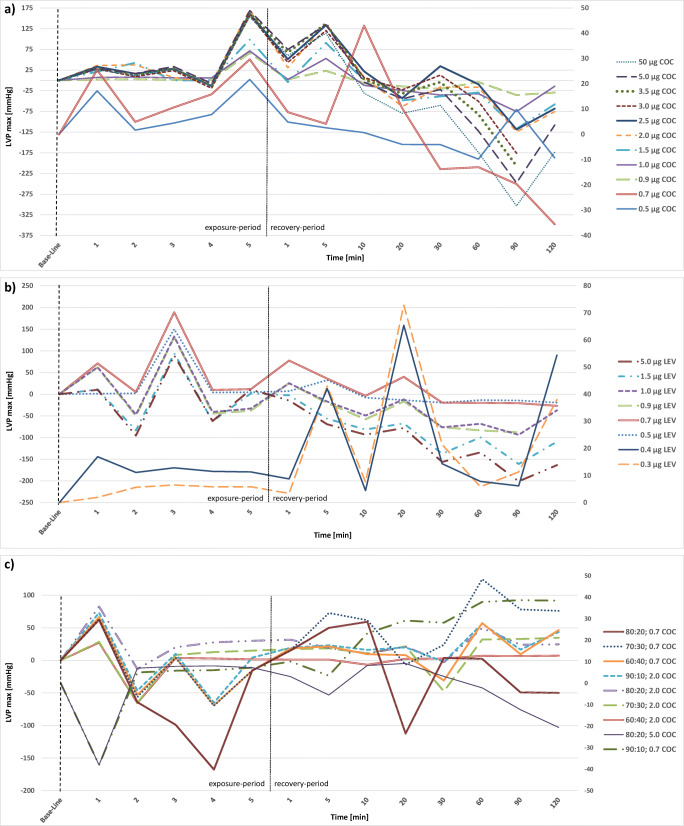
Alterations of left ventricular pressure (LVPmax) in study hearts, presented as stacked line charts: **a** cocaine hearts (COC), **b** levamisole hearts (LEV), **c** mixture hearts cocaine: levamisole with either 0.7 μg or 2.0 μg cocaine (COC). Value “0” is equivalent to the individual base line of each heart after the stabilization period. The first 5 min represent the exposure period, the following minutes 1 to 120 represent the recovery period. Mean values of several individual measurements are shown for a clearer depiction

Hearts in the Langendorff system showed varying maximum left ventricular pressure (LVP_max_), ranging from 33 to 203 mmHg after the stabilization period. Changes of LVP_max_ during the exposure to the evaluated substances and the following recovery period are expressed as the deviation from these individual “base lines” and are presented in Figs. 1 and 3.

LVP_max_ of the hearts of the control group was rather stable, increasing only sligthly from 68 to 74 mmHg during the addition of buffer. Afterwards, LVP_max_ decreased continuously by 10 mmHg. During the administration of ACN, the LVP_max_ increased slightly by 3 to 24 mmHg in the first minute and then decreased during and after the addition until the end of the experiment by 56 to 106 mmHg (Fig. 1b).

In the study group, LVP_max_ of cocaine hearts (Fig. 3a) increased during the exposure period by 4 to 74 mmHg. Most of the hearts also showed a second peak between minute 1 and 20 of the recovery period with an increase of 14 to 56 mmHg. Only the hearts that were exposed to 0.5 μg and 5 μg of cocaine did not show this second peak. Furthermore, the hearts exposed to 5 μg cocaine presented a continuous decrease of LVP_max_ during the recovery-period.

During the exposure to levamisole (Fig. 3b) both an increase of LVP_max_ and a decrease could be observed. The extend of the increase ranged from 4 to 70 mmHg while the decrease ranged from 51 to 57 mmHg. During the recovery period, several hearts showed further “peaks”, meaning an increase of LVP_max_ at different points of time ranging from 6 to 65 mmHg while other hearts presented only little changes or a slight but steady decrease.

LVP_max_ of hearts exposed to a mixture of cocaine and levamisole (Fig. 3c) increased initially during the exposure-period by 2 to 92 mmHg, except two hearts with an immediate decrease of LVP_max_ by 14 mmHg (70:30 mixture with 2 μg cocaine) and 103 mmHg (80:20 mixture with 0.7 μg cocaine). During the recovery period, the overall picture was again very inhomogeneous with temporary increases and decreases of LVP_max_. Overall, LVP_max_ decreased until the end of the period of measurement.

### CF

**Fig. 4:**
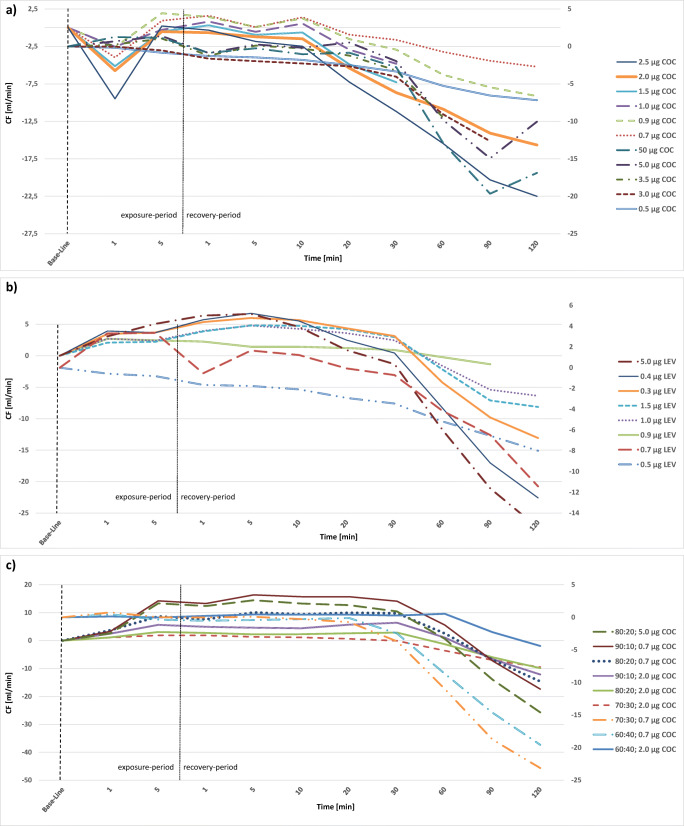
Alterations of coronary flow (CF) in study hearts, presented as stacked line charts: **a** cocaine hearts (COC), **b** levamisole hearts (LEV), **c** mixture hearts cocaine: levamisole with either 0.7 μg or 2.0 μg cocaine (COC). Value “0” is equivalent to the individual base line of each heart after the stabilization period. The first 5 min represent the exposure period, the following minutes 1 to 120 represent the recovery period. Mean values of several individual measurements are shown for a clearer depiction

Hearts in the Langendorff system showed varying CF ranging from 11.56 to 30.45 mL/min after the stabilization period. Changes of CF during the exposure to the evaluated substances and the following recovery period are expressed as the deviation from these individual “base lines” and are presented in Figs. 1 and 4.

CF of the control-hearts was rather stable during the exposure to buffer and ACN, accounting for the time of the administration and the following 30 min. Afterwards, a distinct decrease was seen between 7.7 and 10.5 mL/min (Fig. 1c).

CF of hearts exposed to 1 μg, 1.5 μg, 2 μg and 2.5 μg cocaine showed a decrease during the exposure-period by 0.5 to 4 mL/min and recovered until minute 5 (Fig. 4a). Hearts exposed to 0.9-μg, 3-μg, 3.5-μg, 5-μg and 50-μg cocaine on the other hand presented an increase of CF during the exposure-period by 0.4 to 1.5 mL/min. Afterwards, CF was stable for another 30 min before it started to decrease again by 3 to 7 mL/min until the end of the experiment. The hearts exposed to 0.5 μg cocaine showed a steady decrease of CF of 3.35 mL/min from the beginning until the end of the experiment. The two hearts that were exposed to 0.7 μg cocaine were the only cases in which measurements of one parameter under the influence of the same quantity of one substance differed: one heart presented a decrease of CF during the exposure-period by 5.3 mL/min, while CF of the other heart increased by 0.46 mL/min, followed by unsteady values until minute 10 and a subsequent decrease.

Levamisole treated hearts (Fig. 4b) showed a slight increase of CF by 0.1 to 4.2 mL/min during the exposure-period with the exception of two hearts that presented a decrease of 0.6 (1.5 μg levamisole) and 0.82 mL/min (0.5 μg levamisole). Afterwards CF dropped slowly until the 30th minute and then sharply by 1.14 mL/min to 2.94 mL/min in total. Only for the heart exposed to 0.9-μg levamisole, a final increase was noticed.

In the hearts exposed to a mixture of cocaine and levamisole (Fig. 4c), CF was rather stable (− 0.4 to + 2.5 mL/min) during the exposure-period. Afterwards CF decreased slowly until the end of the experiment by 2.17 mL/min to 7.73 mL/min in total. Only the hearts exposed to the 80:20 mixture with 5 μg cocaine presented an continuous decrease of CF from the beginning to the end of the experiment by 9.67 mL/min.

### H&E staining

**Fig. 5:**
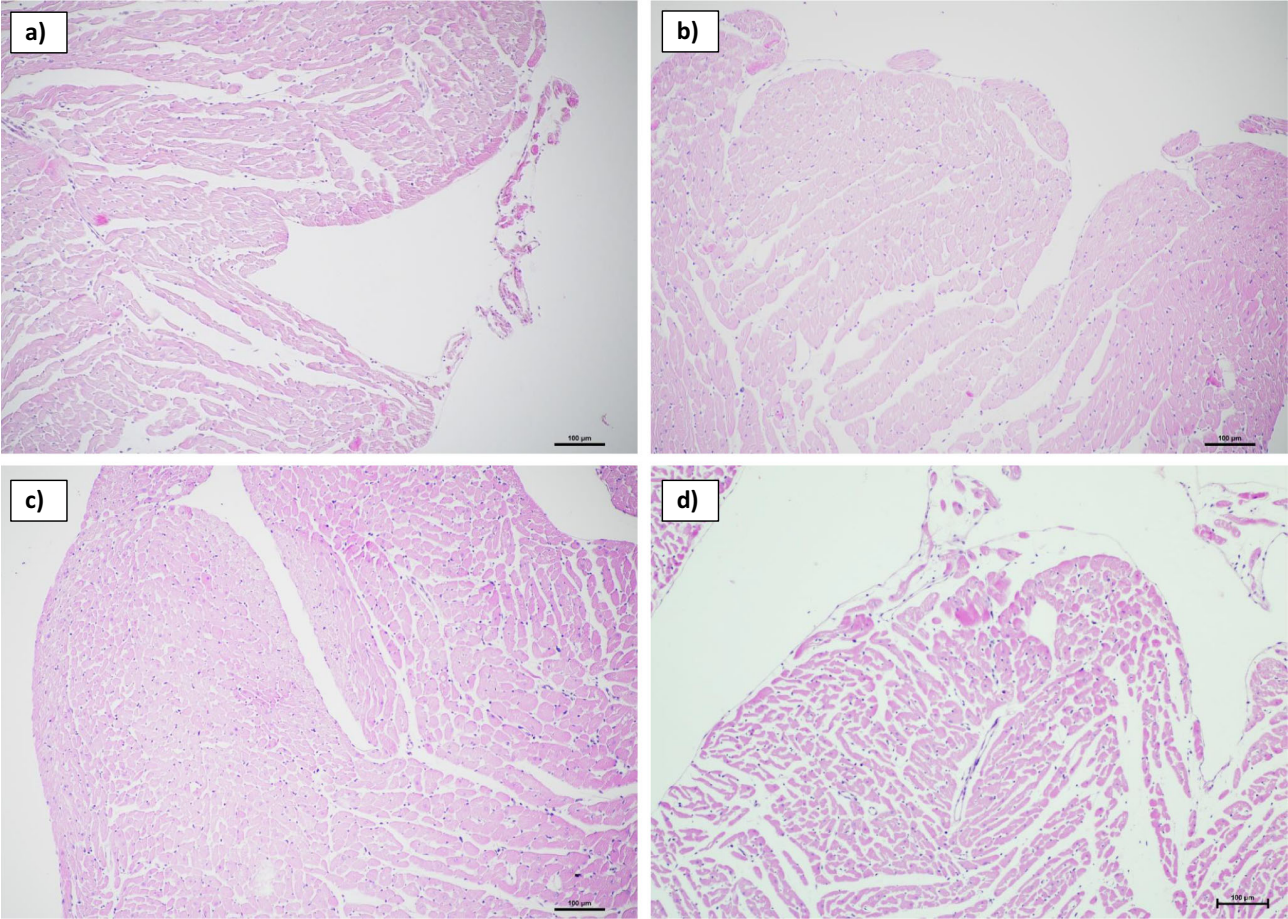
H&E staining results (examples, 100-fold magnification): Sections of the left chamber of three study hearts and one control heart with discrete hypereosinophilic staining of single cells **a**) study heart exposed to 5 μg cocaine, **b**) study heart exposed to 0.9 μg levamisole **c**) study heart exposed to a mixture of cocaine and levamisole at a ratio of 60:40 with 0.7 μg cocaine, **d**) control heart exposed to an additional amount of 0.1 mL acetonitrile over 5 min

Tissue sections of all hearts showed very discreet signs of ischemia, namely hypereosinophilia of single cells located subendocardial in the left ventricle. No differences were seen between study hearts and hearts of the control group. Examples are shown in Fig. 5.

## Discussion

In the present study, we wanted to evaluate if the adulterant levamisole alters, maybe even enhances the cardiac effects of cocaine. We worked with an animal model, the isolated perfused Langendorff heart, and exposed post mortem harvested hearts of rats to different quantities of cocaine, levamisole and a mixture of these two substances while monitoring the functional parameters heart rate, cardiac flow and left ventricular pressure. This observing approach was chosen with a view to unclear cases of death in forensic routine work that are related to a consumption of only low amounts of cocaine.

The results of the hearts of the control group show that administration of ACN or an extra amount of buffer solution alone over 5 min (exposure-period) has some effects on the functional parameters monitored throughout the experiments. However, the range of these alterations was smaller than the ones observed when hearts were exposed to cocaine and/or levamisole. Therefore, the results of the study hearts can be attributed to effects of the two substances on the heart function and are not merely derived from the experimental setup.

The results of the hearts that were exposed to cocaine comply with cardiac effects of cocaine that have been published before [23–25]. For example, our experiments came up with a decrease of heart rate and coronary flow as it was also described by Simkhovich et al. [23] and Vitullo et al. [24]. A decrease of coronary flow and an initial increase of LVP_max_ with a subsequent decrease until the end of the experiment also goes along with the findings of Simkhovich et al. [23]. As the underlying mechanisms, a block of potassium and sodium channels, resulting in systolic and diastolic dysfunction, arrhythmias and a decrease of myocardial contractility and ejection fraction has been described [25]. Cocaine stimulates the release of endothelin-1 which causes vasoconstriction in endothelial cells and inhibits the production of vasodilating nitric oxide [25], resulting in coronary artery spasms that can explain the CF-decrease in our experiments [24]. Furthermore, the decrease of heart rate in cocaine hearts during the exposure period, with the exception of hearts exposed to 1 μg and 2 μg cocaine (see Fig. 2), might be connected to the fact that cocaine blocks fast sodium channels and stabilizes the axonal membrane [26]. With a view to heart rate, severe bradycardia is not uncommon in cocaine users according to Mahoney et al. [27].

Levamisole inhibits monoamine oxidase and has agonistic effects on nicotinic receptors [28] in nematodes and mammalian muscles [29]. In our experiment, levamisole triggered an increase of LVP_max_ during the exposure period in some cases which might be caused by a block of open acetylcholine receptors resulting in a calcium influx [12]. This mechanism might also be responsible for an increase of blood pressure and cases of pulmonary hypertension [14, 18, 30] that had been observed under the treatment of patients with levamisole. In our experiments, there was a slight increase of coronary flow and a deceleration of heart rate while hearts were exposed to levamisole. In the recovery period, the heart rate accelerated, seemingly dose dependent, for at least 5 minutes (0.5 μg levamisole) up to 120 min (5 μg levamisole). The interpretation of this effect is challenging as it suggests that levamisole remains in the myocardium even after it has been administered or triggers a reaction in the cells/in the tissue leading to a long lasting effect [19, 31]. However, research findings are not clear when it comes to the question if cardiovascular effects of levamisole in humans are caused by the substance itself or by the metabolite aminorex. Therefore, it has to be acknowledged that our experiments clearly show alterations of the cardiac function parameters during and after the administration of levamisole, but the underlying mechanism, however, cannot be explained comprehensively.

Regarding the experiments with mixtures of cocaine and levamisole, we observed a deceleration of heart rate during exposure to the substances similar to the one provoked by cocaine alone, followed by an acceleration for up to 90 min that resembled the effect of levamisole as a pure substance. CF of mixture hearts showed only little changes, as if the substances nullified each others’ effect in this respect. LVP_max_ increased but only in the same range as it did under the influence of the individual substances. This means that our experiments did not reveal an exponentiation but rather a combination of the cardiac effects of cocaine and levamisole. No obvious aggravation of certain effects and no emergence of new effects were observed.

When evaluating the results of our experiments, it has to be taken into account that we worked with healthy (rats’) hearts that were not habituated to the exposure of cocaine and levamisole. The function of these hearts was influenced by even low amounts of cocaine and levamisole in several ways. Long-term cocaine consumers might on the one hand be accustomed to the effects of these substances. On the other hand, they typically present chronic heart diseases like coronary artery sclerosis or cardiomyopathy; even more, when cocaine consumption goes along with the abuse of other drugs like marihuana, heroin, alcohol and/or tobacco [6, 32–34], which is quite common. Under such conditions, the risk for fatal cardiac complications triggered by cocaine intake is even higher. Additionally, there is an individual disposition concerning the sensitivity for drug (side) effects. This can be explained by genetic polymorphisms [35]. Numerous studies were able to show that the effects of drugs depend on several genes [36–39]. Referring to cocaine, genes which encode for dopamine transporters [37] or dopamine receptors [36] seem to play the most important role. They may modulate drug sensitivity [36] and reward consuming drugs when dopamine receptors work deficient or an inefficient dopamine system is present [39]. Thus, some persons are especially sensitive and show drug-related (cardiac) effects even when consuming relatively low doses of cocaine. With a view to forensic case work this means that death as a consequence of cocaine consumption does not necessarily require the presence of high concentrations of cocaine in the body. If not only cocaine but also the adulterant levamisole is detected, even low concentrations of these substances might have the potential to cause lethal cardiac effects. The chance is even higher, if at the same time morphologically visible heart diseases exist.

In summary, the results of our experiments demonstrate that not only high but also low doses of cocaine have measurable effects on cardiac function. The same accounts for levamisole. A mixture of both substances does not result in an exponentiation but nevertheless in an addition of the effects of the pure substances. This implies that even consumption of relatively small amounts of cocaine adulterated with levamisole might lead to lethal cardiac complications; even more in cases with a genetic disposition for such side effects and/or preexisting cardiovascular pathologies.

Limitations of our study are the rather small number of examined hearts, due to ethical considerations, and the uncertainty regarding the metabolism and the effects of levamisole/aminorex.
